# Reconceptualizing Benchmarks for Residency Training

**DOI:** 10.7759/cureus.1072

**Published:** 2017-03-02

**Authors:** Chandrew Rajakumar

**Affiliations:** 1 Department of Obstetrics & Gynecology, University of Calgary

**Keywords:** competency, performance, residency, training, postgraduate, burnout, 10000, ericsson, safety

## Abstract

Postgraduate medical education (PGME) is currently transitioning to a competency-based framework. This model clarifies the desired outcome of residency training - competence. However, since the popularization of Ericsson's work on the effect of time and deliberate practice on performance level, his findings have been applied in some areas of residency training. Though this may be grounded in a noble effort to maximize patient well-being, it imposes unrealistic expectations on trainees. This work aims to demonstrate the fundamental flaws of this application and therefore the lack of validity in using Ericsson's work to develop training benchmarks at the postgraduate level as well as expose potential harms in doing so.

## Editorial

Postgraduate medical education (PGME) curricula are undergoing major reforms in transitioning from a time-dependent model with annual advancement of responsibilities to a more learner-centric version in which educational milestones measure progression [1]. Competency-based training petitions educators to quell personal inclinations that trainees require similar amounts of experience to achieve competence in a given component of practice. It seems intuitive that learners will assimilate and consolidate experiences in the cognitive, affective, and psychomotor domains at individual rates. For some, surgical/procedural maneuvers may come intuitively within the first few attempts after which they may excel while others with differing aptitudes may struggle for some time before achieving satisfactory performance and yet, standard metrics such as time, case volume, and patient outcomes are pervasive in residency training. Statements such as “you have not done enough *insert procedure* until you’ve had an *insert complication*” is not uncommon amongst surgeons. This feedback may imprint on trainees and propagate the concept that experience outweighs expertise. Denouncing this perception has been made all the more difficult since the adaptation of Ericsson’s “10,000-hours rule” as a benchmark in PGME [2-4].

Ericsson’s observations were made through investigating the practice patterns of world-class musicians, chess players, typists, athletes, etc. A distillation of his work is that mastering a skill can be achieved if an individual dedicates 10,000 hours to deliberate practice in that particular domain. This formula depicts elite performance as a function of time and quality of practice. This manner of training differs from repetition. Deliberate practice is goal directed and requires focused, dedicated engagement [5].

Patient outcomes are a major concern to physicians. Despite understanding that these are the result of multiple variables interacting in complex systems, many physicians conceptualize poor outcomes as a reflection of the quality of care provided and/or their team, including themselves. The desire to improve one’s self and future generations of physicians to better the well-being of others is noble and this is the admirable context which envelops the aspiration of medical educators to see their pupil turn master. Herein lies the rationale to push residents to achieve 10,000 hours of practice during residency [2-4]. However, the noble goal of educators and trainees is incongruent with that of residency training programs. The product of a PGME curriculum is a competent and safe physician who is capable of practicing medicine within the scope of their training independently. Competence and mastery are dissimilar levels of proficiency and it is the latter to which the 10,000-hours rule applies [5].

Many countries are transitioning their PGME programs to a competency-based medical education (CBME) model. The Royal College of Physicians and Surgeons of Canada supports this by projecting that CBME will ultimately enhance patient care. This transition re-conceptualizes training to progression based on proficiency in milestones grounded in societal and patient needs. CBME allows for more flexible and individualized training plans in which learners are empowered and accountable for their education. The CBME reformation clarifies the ultimate goal of residency training – competence. 

Medical education is a continuum from the undergraduate level through independent practice. At the completion of residency, a physician is at least minimally competent. This level of ability is the summation of minimal proficiency in each competency of that specialty. These competencies are observable and evaluable. Competence of the learner is determined through regular formative assessment throughout their training. Satisfactory performance in all competencies demonstrates the readiness to succeed their training program and although the goal of minimal competence may not be the ultimate aspiration of the physician, it is a necessary stepping-stone on their journey. 

It would be unrealistic to believe that residents can master the broad scope of their specialty. However, during independent practice and life-long learning, elite performance can be cultivated. Graduates focus their care to reflect their passions and the needs of their communities. They may focus on an organ or organ system, particular procedures or techniques or an exact disease.  With time, proficiency in underutilized competencies will decline, while those to which particular attention is paid will progress. This presents a dichotomy between general proficiency and mastery. The relatively general education during residency further affirms the inapplicability of the 10,000-hours rule to PGME. In fact, under the assumption of an 80-hour work week, it would require at least 2.5 years of deliberate practice per competency to master a specialty, during which engagement would be constantly challenged by call-shifts, administrative, scholarly, social, financial, and other commitments.

To date, the body of evidence does not accurately depict the amount of deliberate practice needed to achieve competence in a domain of medicine and further study is required. These data will likely follow successful integration of CBME frameworks and will provide the substrate for evidence-based curriculum design.

In my opinion, it is important to dissuade medical educators from applying the 10,000-hours rule to residency education. The target is unrealistic and differs from training objectives. Thus, it may skew the perspective of teachers by introducing anchoring or focalism and resulting in projection bias during learner assessment and program evaluation. These unattainable expectations could contribute to resident burnout and disengagement through its influences on one's work-life balance. It is important to refocus on the objectives and competencies of one’s specialty and make these requirements transparent. Ten thousand hours of deliberate practice does not apply to postgraduate residency training.
